# Induction of 3-hydroxy-3-methylglutaryl-CoA reductase mediates statin resistance in breast cancer cells

**DOI:** 10.1038/s41419-019-1322-x

**Published:** 2019-01-28

**Authors:** Andy Göbel, Dorit Breining, Martina Rauner, Lorenz C. Hofbauer, Tilman D. Rachner

**Affiliations:** 1 Division of Endocrinology, Diabetes, and Bone Diseases, Department of Medicine III, Technische Universität Dresden, Dresden, Germany; 2 German Cancer Consortium (DKTK), Partner Site Dresden and German Cancer Research Center (DKFZ), Heidelberg, Germany; 3 Center for Healthy Aging, Technische Universität Dresden, Dresden, Germany

## Abstract

The mevalonate pathway has emerged as a promising target for several solid tumors. Statins are inhibitors of the 3-hydroxy-3-methylglutaryl-CoA reductase (HMGCR), the rate-limiting enzyme of this pathway, and are commonly used to treat patients with hypercholesterolemia. Pleiotropic antitumor mechanisms of statins have been demonstrated for several human cancer types. However, cancer cells differ in their individual statin sensitivity and some cell lines have shown relative resistance. In this study we demonstrate, that the human breast cancer cell lines MDA-MB-231, MDA-MB-468, MCF-7, and T47D are differentially affected by statins. Whereas the vitality of MDA-MB-231 and MDA-MB-468 cells was reduced by up to 60% using atorvastatin, simvastatin, or rosuvastatin (*p* < 0.001), only marginal effects were seen in T47D and MCF-7 cells following exposure to statins. Statin treatment led to an upregulation of HMGCR mRNA and protein expression by up to sixfolds in the statin-resistant cells lines (*p* < 0.001), but no alterations of HMGCR were observed in the statin-sensitive MDA-MB-231 and MDA-MB-468 cells. The knockdown of HMGCR prior to statin treatment sensitized the resistant cell lines, reflected by a 70% reduction in vitality, increased apoptotic DNA fragmentation (sixfold) and by accumulation of the apoptosis marker cleaved poly-ADP ribose polymerase. Statins induced a cleavage of the sterol-regulatory element-binding protein (SREBP)-2, a transcriptional activator of the HMGCR, in T47D and MCF-7 cells. The inhibition of SREBP-2 activation by co-administration of dipyridamole sensitized MCF-7 and T47D cells for statins (loss of vitality by 80%; *p* < 0.001). Furthermore, assessment of a statin-resistant MDA-MB-231 clone, generated by long-term sublethal statin exposure, revealed a significant induction of HMGCR expression by up to 12-folds (*p* < 0.001). Knockdown of HMGCR restored statin sensitivity back to levels of the parental cells. In conclusion, these results indicate a resistance of cancer cells against statins, which is in part due to the induction of HMGCR.

## Introduction

Breast cancer remains one of the leading causes of cancer deaths in women with more than half a million deaths per year worldwide^1^. The occurrence of local relapse or distant metastases is a common problem. In addition, relapsing tumors often show de novo resistances towards standard therapies and are difficult to treat^2^. New therapeutic targets are currently subject to ongoing research. Recently, the mevalonate pathway has emerged as a promising therapeutic candidate in several malignancies including melanoma, prostate, and breast cancer^3,4^. This complex pathway is best known for its role in the production of cholesterol. Mevalonate is the basic intermediate substrate for the subsequent synthesis of isoprenoids such as cholesterol^5^. Among others, additional end products of the pathway are farnesyl pyrophosphate and geranylgeranyl pyrophosphate which are necessary for post-translational modifications of many proteins, a process referred to as protein prenylation^6^. The rate-limiting enzyme of the mevalonate pathway is the 3-hydroxy-3-methylglutaryl-CoA reductase (HMGCR), which converts HMG-CoA to mevalonate and is blocked by statins. This protein and the associated cholesterol production are tightly controlled, both by several transcriptional and post-translational regulatory mechanisms^7^.

It is well-accepted that the mevalonate pathway drives malignant transformation. Treatment of tumor cells in vitro or of melanoma-bearing mice with mevalonate accelerates tumor proliferation and growth^8^. Also, ectopic expression of the HMGCR increases tumor growth of the subcutaneously injected human liver carcinoma cell line HepG2, suggesting that the enzyme acts as an oncogene. HMGCR expression is also associated with a poor outcome in breast cancer patients^9^. In clinical breast cancer samples a poor outcome has been observed in those carrying a mutant form of p53 that increases the activity of the mevalonate pathway^10^. In addition, cancer cells profoundly rely on several of the mevalonate pathway products^8,11^. More than a century ago, the accumulation of cholesterol crystals was first observed in tumor specimen^12^ and more recently, a positive correlation between cholesterol and cancer risks has been shown for various human malignancies. In melanoma, patients survival is decreased when cancer cells show an enhanced expression of cholesterol synthesis genes^13^. Elevated cholesterol is considered as a risk factor for breast cancer and one of its primary metabolites, the estrogen receptor (ER)-ligand 27-hydroxycholesterol, increases tumor growth and metastasis in murine models of ER-positive breast cancer^14,15^. Further studies have revealed a pivotal role of geranylgeranylation for the maintenance of breast cancer stem cell populations^16^.

Statins are well-established drugs used to lower serum cholesterol in patients^17^. They act by inhibiting the HMGCR. Several studies show that statins exert antitumor effects in human malignancies, including breast cancer^18–20^. However, other studies have failed to show any meaningful effect^4^. Preclinical and clinical studies in breast cancer have yielded varying effects of statins depending on the cell lines and cohorts, respectively^21–24^. It remains unclear why certain breast tumors are more susceptible to statin treatment than others and current efforts are made to identify biomarkers that would predict tumor statin sensitivity^25^. In this study we aimed at identifying the underlying mechanisms of statin resistance in breast cancer.

## Results

### MCF-7 and T47D breast cancer cells are more resistant to statin treatment than MDA-MB-231 cells

**Fig. 1:**
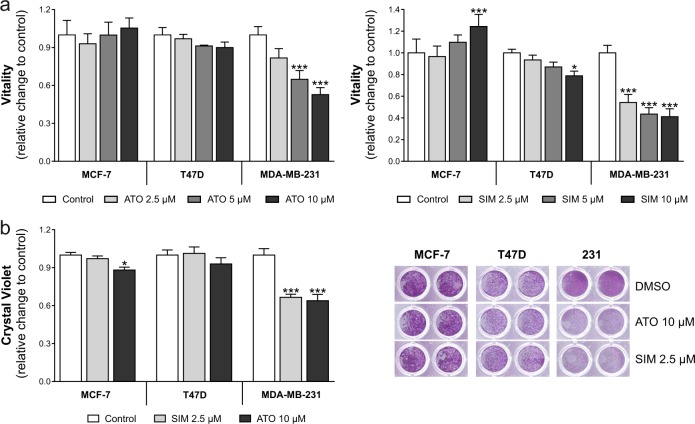
Human breast cancer cells vary in their sensitivity to atorvastatin (ATO) and simvastatin (SIM). **a** MDA-MB-231, MCF-7, and T47D cells were treated with increasing concentrations of SIM and ATO for 48 h. The impact on cell vitality was measured using the CellTiterBlue^®^ assay. **b** Adherent breast cancer cells were stained with crystal violet after treatment with SIM (2.5 µM) and ATO (10 µM) for 48 h. Absorbance was measured at 595 nm after elution with 10% SDS. Data are shown as mean ± SD of at least three individual experiments. (*p ≤ 0.05; ***p ≤ 0.001)

Breast cancer cell lines were treated with increasing concentrations of atorvastatin and simvastatin (Fig. 1a). Cell vitality was reduced by 50–60% in MDA-MB-231 cells at a concentration of 10 µM (*p* < 0.001). By contrast, vitality of MCF-7 cells and T47D cells was not affected by up to 10 µM of atorvastatin. Cell vitality increased (+20%, *p* < 0.05) in MCF-7 cells upon 10 µM simvastatin and was reduced in T47D cells (−20%, *p* < 0.05). These observations were confirmed by crystal violet staining, where simvastatin (2.5 µM) or atorvastatin (10 µM) reduced the number of viable cells by 40% in MDA-MB-231 cells (Fig. 1b; *p* < 0.001) but had little or no effect on MCF-7 and T47D cells. These results show a relative resistance of MCF-7 and T47D cells to statin treatment.

### Statin treatment induces HMGCR gene and protein expression in MCF-7 and T47D breast cancer cells

**Fig. 2:**
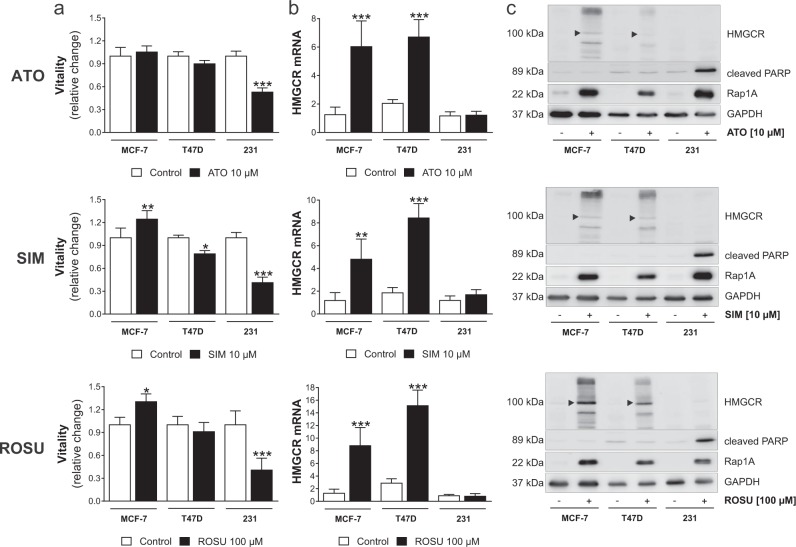
Statin treatment increases 3-hydroxy-3-methylglutaryl-CoA (HMGCR) gene and protein expression in statin-resistant breast cancer cell lines. Human MCF-7, T47D, and MDA-MB-231 breast cancer cells were treated with atorvastatin (ATO), simvastatin (SIM), or rosuvastatin (ROSU) for 48 h. Cell vitality was measured using the CellTiterBlue^®^ assay (**a**). Gene expression of the HMGCR was assessed using quantitative real-time PCR. Glyceraldehyde-3-phosphate dehydrogenase (GAPDH) was used a housekeeper control (**b**). Protein expression of HMGCR, cleaved poly-ADP ribose polymerase (PARP) and Rap1A was investigated using western blot analysis (**c**). Representative images are shown. The black arrowhead indicates the ~100 kDa size as the proposed protein size of the HMCGR. The additional bands point towards several isotypes and/or glycosylation status of the protein. Data are shown as mean ± SD of at least three individual experiments. (*p ≤ 0.05; **p ≤ 0.01; ***p ≤ 0.001)

As statin sensitivity varied among the investigated cell lines, we next assessed the gene expression of the HMGCR, the statin targeting enzyme. First, vitality of the three breast cancer cell lines after exposure to high simvastatin, atorvastatin, and rosuvastatin concentrations was directly compared (Fig. 2a). Interestingly, while MCF-7 and T47D cell vitality was not reduced upon statin treatment, HMGCR gene expression was significantly induced by up to eightfolds (Fig. 2b; *p* < 0.001). By contrast, HMGCR expression of statin-sensitive MDA-MB-231 cells remained unchanged after statin exposure (Fig. 2b). Of note, no significant difference of baseline HMGCR mRNA expression was observed between the three cell lines (polymerase chain reaction (PCR) threshold cycles were 23.3 ± 1.0 for MDA-MB-231, 23.3 ± 1.2 for MCF-7, and 23.6 ± 0.7 for T47D cells).

Our observations were confirmed at protein level (Fig. 2c). All statins inhibited the mevalonate pathway as shown by the accumulation of unprenylated Rap1A. Statin resistance of T47D and MCF-7 cells was confirmed by assessment of cleaved poly-ADP ribose polymerase (PARP) as a marker of apoptosis, which increased in MDA-MB-231 cells but not in T47D and MCF-7 cells after statin exposure. HMGCR protein accumulated in statin-insensitive MCF-7 and T47D cells, but not in MDA-MB-231, when cells were treated with high statin concentrations. Notably, the HMGCR protein appeared as several bands with different molecular weights. In addition, the observations were confirmed by using an alternative HMGCR-specific antibody (ab214018 from Abcam; Suppl. Fig. 1a).

To analyze if the absence of a HMGCR induction is associated with a statin-sensitive phenotype, we additionally treated human triple-negative MDA-MB-468 breast cancer cells with atorvastatin, simvastatin, and rosuvastatin. Here, vitality and cell number were reduced by up to 45% (Suppl. Fig. 2a; *p* < 0.001). When we analyzed HMGCR mRNA expression 48 h after the treatment with high concentrations of statins, a nonsignificant increase was observed (Suppl. Fig. 2b). To compare all four tested human breast cancer cell lines, we additionally depicted the fold-increase of HMGCR gene expression after statin treatment. Cell lines were arranged on the basis of their statin sensitivity according to the loss of vitality. Here, we were able to show that a higher post-statin HMGCR mRNA induction is accompanied by a decreased statin sensitivity (Suppl. Fig. 2c).

### HMGCR knockdown reverses statin resistance in MCF-7 and T47D breast cancer cells

**Fig. 3:**
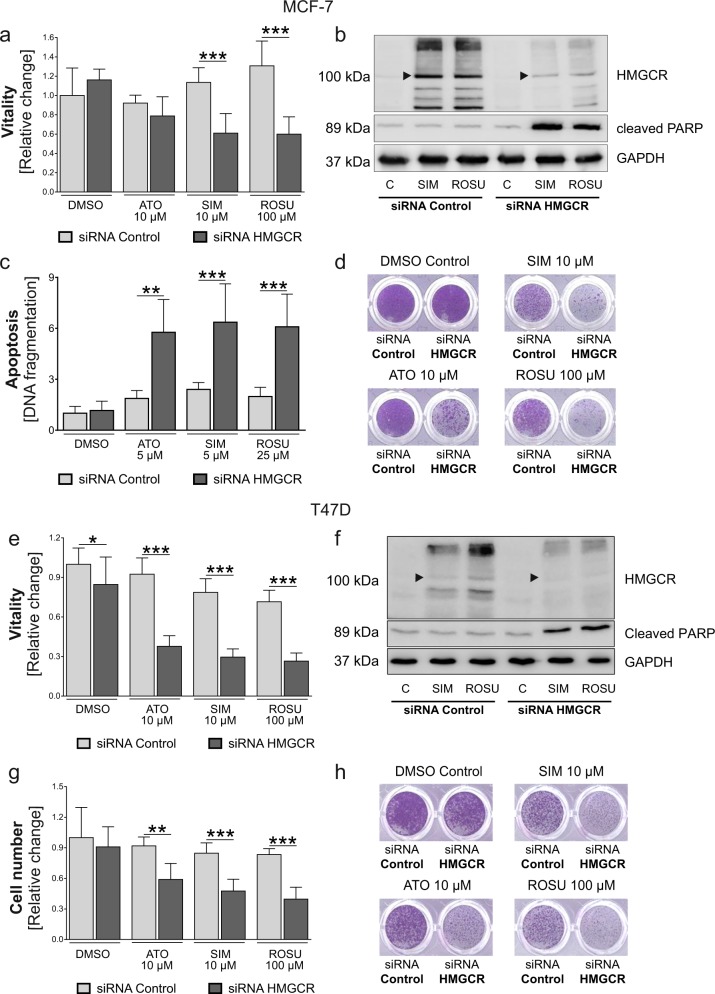
Knockdown of 3-hydroxy-3-methylglutaryl-CoA (HMGCR) sensitizes statin-resistant breast cancer cells to atorvastatin (ATO), simvastatin (SIM), and rosuvastatin (ROSU). Human MCF-7 (**a**–**d**) and T47D (**e**–**h**) cells were treated with HMGCR-specific and control siRNA. Cells were treated with statins 24 h after HMGCR knockdown for 48 h. Cell vitality was measured using the CellTiterBlue^®^ assay (**a**, **e**). Protein expression of HMGCR and cleaved poly-ADP ribose polymerase (PARP) was investigated using Western blot analysis (**b**, **f**). Representative images are shown. The black arrowhead indicates the ~100 kDa size as the proposed protein size of the HMCGR. DNA fragmentation in MCF-7 cells was analyzed using the Cell Death Detection ELISA^Plus^ (**c**). Adherent breast cancer cells were stained with crystal violet after treatment and absorbance was measured at 595 nm after elution with 10% SDS. Data are shown as mean ± SD of at least three individual experiments. (*p ≤ 0.05; **p ≤ 0.01; ***p ≤ 0.001)

Next, HMGCR was knocked-down using small interfering (si)-RNA prior to statin treatment (Suppl. Fig. 1b). HMGCR knockdown had no direct effect on cell vitality in MCF-7 cells. In T47D cells, HMGCR knockdown itself reduced cell vitality by 10% (Fig. 3a, e; *p* < 0.05). However, the knockdown of HMGCR prior to statin treatment significantly sensitized the tumor cells to statins. Cell vitality was reduced by up to 70% in MCF-7 (Fig. 3a) and T47D cells (Fig. 3e) in comparison to control-transfected breast cancer cells (*p* < 0.001). These effects were higher in T47D cells and confirmed by crystal violet staining (Fig. 3d, g, h). In addition, the knockdown of HMGCR prior to statin treatment resulted in apoptosis as seen by accumulation of cleaved PARP, whereas HMGCR protein induction was diminished in both cell lines (Fig. 3b, f). Again, the reduced induction of HMGCR protein upon statin treatment by HMGCR-specific siRNA was verified by using the alternative HMGCR-specific antibody (Suppl. Fig. 1b). In MCF-7 cells we additionally analyzed DNA fragmentation in cells with an intact cell membrane as a sign of apoptosis. In line with previous findings, the combination of any statin with HMGCR knockdown significantly enhanced DNA fragmentation up to sixfold compared to siRNA control (*p* < 0.001; Fig. 3c). Hence, the inhibition of statin-induced HMGCR sensitizes MCF-7 and T47D breast cancer cells to the antitumor effects of statins.

### Targeting the transcriptional activation of the HMGCR sensitizes T47D and MCF-7 breast cancer cells to statins

Next, we aimed to analyze the mechanism of the induction of HMGCR expression in MCF-7 cells after statin exposure. In normal cells, cholesterol can be either obtained by the low-density lipoprotein receptor (LDLR)-mediated uptake or by synthesis via the mevalonate pathway. The pathway is activated by low intracellular sterol levels which drive the cleavage of transcription factors referred to as sterol regulatory element-binding proteins (SREBPs). They bind to sterol-regulatory elements in the promotor region of certain target genes like HMGCR and the LDLR (transcriptional regulation of the HMGCR)^26^. Of the three SREBP isoforms, SREBP-2 plays the most significant role in regulating key genes of cholesterol regulation^7^.

**Fig. 4:**
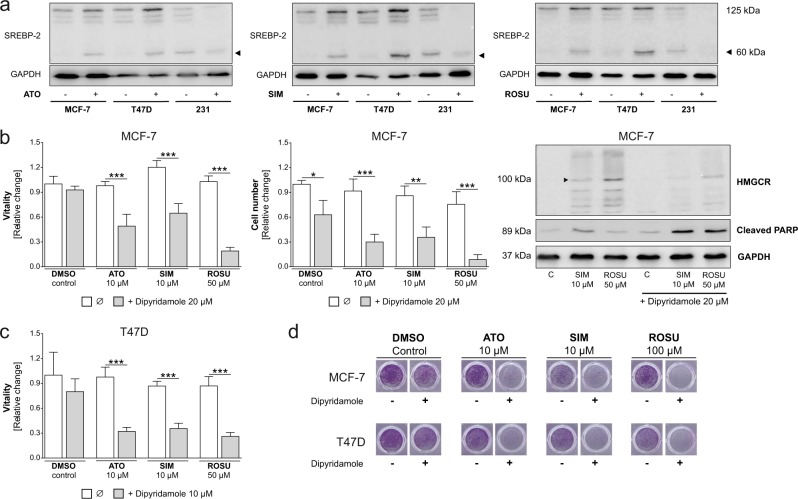
Targeting the transcriptional activation of 3-hydroxy-3-methylglutaryl-CoA (HMGCR) sensitizes statin-resistant breast cancer cells to atorvastatin (ATO), simvastatin (SIM), and rosuvastatin (ROSU). Human MCF-7, T47D and MDA-MB-231 breast cancer cells were treated with different statins. Sterol regulatory element-binding protein 2 (SREBP-2) protein expression was analyzed using Western blot (**a**). MCF-7 and T47D cells were treated with statins and dipyridamole for 48 h. Cell vitality was measured using the CellTiterBlue® assay (**b**, **c**). Adherent breast cancer cells were stained with crystal violet after treatment and absorbance was measured at 595 nm after elution with 10% SDS (**b**, **d**). Protein expression of HMGCR and cleaved poly-ADP ribose polymerase (PARP) was investigated using Western blot analysis. Representative images are shown (**b**). Data are shown as mean ± SD of at least three individual experiments. (*p ≤ 0.05; **p ≤ 0.01; ***p ≤ 0.001)

Treatment with atorvastatin, simvastatin, and rosuvastatin led to an accumulation of cleaved SREBP-2 both in T47D and MCF-7 cells. By contrast, SREBP-2 protein was already cleaved under control conditions and disappeared after statin exposure in MDA-MB-231 (Fig. 4a). As statin-mediated SREBP cleavage also induces LDLR expression as the most important mechanism for driving cholesterol uptake in target cells^26^, we analyzed LDLR mRNA levels in breast cancer cells after statin treatment. LDLR expression was induced by up to threefolds in MCF-7 and T47D cells (*p* < 0.001) by statins. In MDA-MB-231 cells basal LDLR mRNA expression was significantly higher compared to MCF-7 and T47D cells and was suppressed by statins (Suppl. Fig. 3a; *p* < 0.01). Transcriptional upregulation of key genes of the mevalonate pathway was confirmed by assessment of the farnesyl diphosphate synthase (FDPS), a downstream enzyme of the HMGCR, which was significantly induced by statins in MCF-7 and T47D cells (*p* < 0.001), but remained unaltered in MDA-MB-231 cells (Suppl. Fig. 3b).

The contribution of SREBP-2 to statin resistance was further confirmed using dipyridamole, an antiplatelet agent and known inhibitor of SREBP-2 cleavage^27^. Here, treatment of MCF-7 cells with atorvastatin, simvastatin, or rosuvastatin alone had no impact on cell vitality. However, when combined with 20 µM dipyridamole, vitality and cell number were significantly suppressed by up to 80% (*p* < 0.001). Western blot analysis confirmed induction of HMGCR upon simvastatin and rosuvastatin treatment which was impaired by co-administration of dipyridamole while cleaved PARP accumulated (Fig. 4b). The results were confirmed for T47D cell vitality (Fig. 4c) and by crystal violet staining in both cell lines (Fig. 4d; representative pictures). These results support the hypothesis that statin resistance in MCF-7 and T47D breast cancer cells is mediated by a regulatory feedback loop via the HMGCR that counteracts the inhibition of the mevalonate pathway.

### Establishment of a statin-resistance in MDA-MB-231 cells by reactivation of the HMGCR regulatory feedback loop

**Fig. 5:**
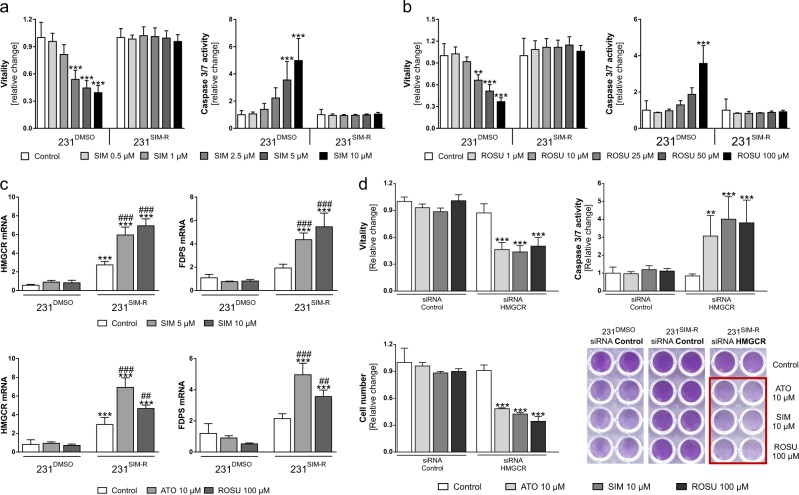
Statin-sensitive MDA-MB-231 cells gain statin-resistance during long-term simvastatin treatment by activating the 3-hydroxy-3-methylglutaryl-CoA (HMGCR). Statin resistant (231^SIM-R^) and control (231^DMSO^) MDA-MB-231 cells were treated with simvastatin (SIM) and rosuvastatin (ROSU) for 48 h. Vitality and caspase 3/7 activation were measured using the CellTiterBlue^®^ and Caspase 3/7 Glo^®^ assay (**a**, **b**). Statin resistant (231^SIM-R^) and control (231^DMSO^) MDA-MB-231 cells were treated with SIM, ROSU, or atorvastatin (ATO) and gene expression of HMGCR and farnesyl diphosphate synthase (FDPS) analyzed using quantitative real-time PCR (**c**). Statin resistant (231^SIM-R^) MDA-MB-231 cells were transfected using HMGCR-specific or control siRNA and treated with SIM, ATO, or ROSU after 24 h for 48 h. Vitality and caspase 3/7 activation were measured using the CellTiterBlue^®^ and Caspase 3/7 Glo^®^ assay. Adherent breast cancer cells were stained with crystal violet after treatment and absorbance was measured at 595 nm after elution with 10% SDS (**d**). Data are shown as mean ± SD of at least three individual experiments. (*p ≤ 0.05; **p ≤ 0.01; ***p ≤ 0.001 vs. control treatment of 231^DMSO^ cells) (**a**–**c**); ^##^*p* ≤ 0.01; ^###^*p* ≤ 0.001 vs control treatment of 231^SIM-R^ cells (**c**); (**p ≤ 0.01; ***p ≤ 0.001 vs. control treatment of siRNA HMGCR transfected 231^SIM-R^ cells (**d**))

Having shown that MCF-7 and T47D cells can be sensitized to statins by targeted suppression of the HMGCR-mediated regulatory feedback loop, we aimed to investigate if MDA-MB-231 cells are able to adapt this principle following long-term selection pressure (see Methods section and Suppl. Fig. 4a). The established simvastatin-resistant MDA-MB-231 subclone is referred to as 231^SIM-R^. MDA-MB-231 cells with long-term DMSO treatment were used as the parental control (231^DMSO^). A time-course experiment of untreated MDA-MB-231, 231^DMSO^, and 231^SIM-R^ cells revealed no differences in the growth potential (increase of vitality) of 231^DMSO^ cells compared to unselected MDA-MB-231 cells (Suppl. Fig. 4b). Basal 231^SIM-R^ cell vitality was slightly reduced over the time (*p* < 0.001). 231^DMSO^ and 231^SIM-R^ cells were treated with increasing concentrations of simvastatin (0.5–10 µM). Here, vitality was lost by up to 60% and apoptosis induced by fivefold in 231^DMSO^ cells (*p* < 0.001) while 231^SIM-R^ cells were resistant to the treatment (Fig. 5a). Of note, resistance of the cells was not restricted to simvastatin, but also to rosuvastatin (Fig. 5b; *p* < 0.001). When treating 231^DMSO^ and 231^SIM-R^ cells with simvastatin, atorvastatin, or rosuvastatin, we not only observed a significantly increased baseline HMGCR expression in 231^SIM-R^ cells but were also able to demonstrate, that these resistant cells responded to statin treatment with a significant further upregulation of HMGCR by up to 12-folds (Fig. 5c; *p* < 0.001). The upregulation of the mevalonate pathway in 231^SIM-R^ cells was confirmed by showing that FDPS expression was significantly increased by any of the statins. Next, we knocked-down the HMGCR prior to statin treatment using specific siRNA. Control-transfected 231^SIM-R^ cells did not respond to atorvastatin, simvastatin, or rosuvastatin. By contrast, HMGCR knockdown sensitized 231^SIM-R^ cells to any statin as demonstrated by loss of vitality and cell mass by 50%, increase of caspase 3/7 activation by up to fourfold, and accumulation of cleaved PARP (Fig. 5d; *p* < 0.001 and Suppl. Fig. 7). The efficacy of the knockdown was validated by real-time PCR (Suppl. Fig. 5a, b): First, baseline expression of HMGCR was reduced by 58% (*p* < 0.05) using the HMGCR-specific siRNA. In addition, the experiments confirmed the induction of HMGCR mRNA expression in 231^SIM-R^ cells upon treatment with any statin by up to 2.6-fold (*p* < 0.001). However, no induction of HMGCR was seen when cells had been pretransfected with HMGCR-specific siRNA.

Moreover, the sensitization of 231^SIM-R^ cells for statins by HMGCR knockdown was demonstrated by the analysis of the expression of two antiapoptotic genes, B-cell lymphoma 2 (*BCL-2*), and survivin (*SVV*). We have previously shown that these genes are suppressed when targeting the mevalonate pathway^28^. No alteration was observed in statin treated and control-transfected 231^SIM-R^ cells. HMGCR knockdown moderately increased baseline expression of both genes and concomitant treatment with any statin significantly suppressed both *BCL-2* and *SVV* gene expression by up to 80% (Suppl. Fig. 5; *p* < 0.001).

We also transfected parental 231^DMSO^ cells with HMGCR-specific siRNA and treated them with all three statins (Suppl. Fig. 6). In control conditions, the cells showed a significant loss of vitality and the induction of activated caspases 3 and 7 (*p* < 0.001). These effects were significantly potentiated when HMGCR protein was not only inhibited by statins but also suppressed on mRNA level by the specific siRNA (final induction of apoptosis up to tenfold; *p* < 0.001).

In addition, in 231^SIM-R^ cells, SREBP-2 protein was cleaved under control conditions and remained in the activated cleaved form when cells were treated with statins. However, HMGCR knockdown increased the accumulation of cleaved SREBP-2, whereas co-administration of statins led to a diminished SREBP-2 signal; the same reaction which was observed in statin-sensitive MDA-MB-231 cells (Suppl. Fig. 7 and Fig. 4a). These results demonstrate that statin-sensitive MDA-MB-231 cells gain a statin-resistant phenotype by long-term simvastatin treatment that is mediated by a constitutive increase in HMGCR expression and can be reversed by targeting the HMGCR.

## Discussion

The inhibition of the mevalonate pathway by statins provokes pleiotropic antitumor effects in preclinical settings, including breast cancer^19,29,30^. However, statin sensitivity of cancer cell lines varies and ambiguous results have been obtained by clinical trials^25^.

In this study, we demonstrate that atorvastatin, simvastatin, and rosuvastatin induced a significant loss of vitality in human triple-negative MDA-MB-231 and MDA-MB-468 breast cancer cells, although antitumor effects in MDA-MB-468 cells were less pronounced compared to MDA-MB-231 cells. In this regard it may be important to mention that the molecular classification of these cell lines is different even sharing the triple hormone receptor negativity. Whereas MDA-MB-231 cells represent the claudin-low subtype, MDA-MB-468 cells are classified as “basal”^31^.

Ten micrometer of simvastatin were more efficient than the same amount of atorvastatin and tenfold of that concentration was necessary to obtain similar results with rosuvastatin. This response of MDA-MB-231 cells is in line with a previous report on the IC_50_ values of several statins in these cells^32^. This statin-induced loss of vitality in T47D cells was only seen for high-simvastatin concentrations and was absent in MCF-7 cells. The varying simvastatin sensitivity of these cell lines was demonstrated in other studies^33^. Similar observations have been made in a study, where MCF-7 cells were much less sensitive to fluvastatin, simvastatin, and lovastatin compared to MDA-MB-231 cells^21^. A further report demonstrated different susceptibilities of human breast cancer cell lines to fluvastatin. Here, statin sensitivity was associated with a basal-like, ER-negative phenotype of the tumor cells and hormone receptor-positive cells were less sensitive^22^. In a perioperative trial, patients were treated with fluvastatin 3–6 weeks before surgery and tumor tissue was analyzed for markers of proliferation and apoptosis. Although not significant, ER-negative high-grade tumors appeared more susceptible evidenced by higher loss of the Ki67 proliferation marker and increased apoptosis^23^.

To investigate the underlying mechanisms of statin sensitivity/resistance, we focused on the regulation of the HMGCR and observed a strong induction in the statin-resistant MCF-7 and T47D cells following statin treatment. The knockdown of the HMGCR prior to statin treatment significantly sensitized MCF-7 and T47D cells to any statin. These findings demonstrate that the induction of HMCGR can mediate statin resistance in breast cancer.

A statin-induced feedback response via induction of HMGCR has been previously described in fungi^5^. In multiple myeloma cells, HMGCR upregulation was also observed in lovastatin-resistant cell lines but was absent in the sensitive ones. Ectopic HMGCR expression decreased the sensitivity of these cell lines^34^. Assessment of primary breast cancer samples revealed that the expression of HMGCR was a predictor of a prolonged recurrence-free survival in ER-positive, but not in ER-negative tumors^35^. In a window-of-opportunity trial, breast cancer patients were treated with atorvastatin 2 weeks before surgery and pre- and post-treatment immunohistochemical staining expression of HMGCR was analyzed. Here, HMGCR staining intensity in tumor samples was significantly stronger in postatorvastatin tumor samples compared to the pretreatment samples. In addition, the loss of Ki67 proliferation index in postatorvastatin tumor samples was higher in ER-negative tumors compared to the ER-positive ones, which is in line with our observation using ER-negative MDA-MB-231 and MDA-MB-468 and ER-positive MCF-7 and T47D cells^24^. In vitro analyses confirmed these observations, in which atorvastatin treatment resulted in an accumulation of HMGCR protein in resistant breast cancer cell lines only^36^.

The final arrangement of all used cell lines on the basis of their statin sensitivity and the poststatin increase in HMGCR expression support our hypothesis that a higher potential of a poststatin HMGCR feedback response is associated with an increased statin resistance.

The use of two different primary HMGCR-specific antibodies revealed weak protein signals in untreated MCF-7, T47D, MDA-MB-468, and MDA-MB-231 cells, although gene expression based on threshold cycle levels appeared high. The HMGCR is a strongly regulated protein. In addition to post-transcriptional regulation via SREBPs, there is a post-translational regulation mediated by the endoplasmic reticulum-associated protein degradation: when cells are endowed with a sufficient amount of sterols and mevalonate pathway products, HMGCR degradation is accelerated and half-life reduced to a few minutes to hours^37^. We assume that basal HMGCR protein levels in the statin-resistant breast cancer cell lines is low under normal conditions with sufficient amounts of mevalonate-derived products. However, upon inhibition of the mevalonate pathway and deprivation from these metabolites such as sterols, HMGCR is induced, resulting from both the described transcriptional and post-translational regulatory processes^38^. However, statin-sensitive breast cancer cells have a disrupted HMGCR feedback loop. Importantly, several signals of different sizes appeared in the cell lines using two individual HMGCR-specific antibodies. We argue that these signals represent different forms of the protein, either carrying different post-translational modifications^7^ or existing in the cleaved status after the described endoplasmic reticulum-associated protein degradation (according to data sheet of sc-271595, the molecular weight of HMGCR C-terminal cleavage products is 40/55 kDa). In addition, the HMGCR transcript can exist in two different forms, a full-length and an alternatively spliced one^39^.

Next, we focused on which pathways mediate the induction of HMGCR expression in statin-resistant MCF-7 and T47D. Statins do not only inhibit the HMGCR but also promote a regulatory feedback response when cholesterol levels are reduced within the cell. This involves the activation of SREBPs and the subsequent upregulation of cholesterol synthesis genes and the LDLR on the cell surface allowing for an accelerated cholesterol uptake from the blood^5,26^. First, SREBP-2 cleavage was observed in T47D and MCF-7 cells after treatment with any statin, whereas the protein was already cleaved in MDA-MB-231 cells under control conditions and disappeared in the presence of statins. A relationship between reduced SREBP-2 expression and an increased statin sensitivity was observed in ovarian cancer cells where oxysterols suppressed SREBP-2 and potentiated the statin antitumor effects^40^. While MCF-7 and T47D cells responded with an increased LDLR expression, MDA-MB-231 cells had higher baseline LDLR expression which was reduced by statins. This is in line with the observed change of SREBP-2 protein cleavage. In previous studies in prostate cancer cell lines, LDLR expression was reduced by simvastatin in sensitive PC-3 cells, but was significantly upregulated in less sensitive LNCaP cells^41^. These results support the relationship between the antitumor effects of statins and a regulatory feedback response via the SREBP-2/HMGCR/LDLR axis which is lacked by statin-sensitive tumor cell lines.

We disrupted this feedback response by combining statins with dipyridamole, a known inhibitor of SREBP-2 cleavage. This approach sensitized both T47D and MCF-7 cells to all three tested statins. Studies in multiple myeloma and leukemia have previously revealed that the combination of dipyridamole and statins induces apoptosis in vitro and reduces the tumor burden in a murine xenograft model^27,42^. Furthermore, breast and lung cancer cells are sensitized to fluvastatin by knockdown of SREBP-2A which abrogates the HMGCR induction^43^.

Finally, we generated a statin-resistant MDA-MB-231 subclone by long-term treatment with high-simvastatin concentrations. In these cells, baseline HMGCR expression was significantly higher compared to the statin-sensitive parental cells and was further increased upon statin treatment. Knockdown of HMGCR re-sensitized cells to statins, indicating HMGCR as a primary mediator of this resistance. Further investigations are necessary to unravel the mechanisms of statin-resistance acquisition in these cells.

Interestingly, a potentiation of statin-sensitivity of parental MDA-MB-231^DMSO^ was also achieved by the combination of statin treatment and HMGCR knockdown. These observations may indicate that the regulatory feedback loop in these statin-sensitive cells has a residual functionality or that the applied statins can more effectively inhibit HMGCR, when its basal expression is already reduced. Hence, double targeting of HMGCR by statins on protein level and siRNA on gene level may be a useful strategy to hit both statin-sensitive and statin-resistant breast cancer cells.

Limitations of our study include the use of in vitro cultures only and the high-statin concentrations. Serum statin concentration in patients ranges from 0.002 to 0.1 µM^19^ and the accumulation within breast tumor tissue remains unclear. Further, it is not clear whether the differences in the statin-induced feedback loop are the main mechanism that defines statin sensitivity in human breast cancer cells. For example, although we did not see differences in basal HMGCR gene expression in MCF-7, T47D, MDA-MB-468, and MDA-MB-231 cells, enzymatic activity may vary among the cell lines. Along those lines, epigenetic regulations of the HMGCR or further mevalonate pathway genes cannot be excluded from a potential contribution to statin-resistance mechanisms^44^. Furthermore we have focused our investigations on changes of the SREBP-2/HMGCR/LDL-R axis in breast cancer cell lines. Future experiments need also to analyze any post-statin alterations on regulators upstream of the SREBP proteins, including the SREBP cleavage-activating protein (SCAP) or Insig-1/2. These proteins are implicated in sterol-sensing and mediating cleavage, transport, and activation of SREBP proteins when sterol levels decline^37^.

In addition, the cells express wild-type (MCF-7) or different mutated forms (T47D, MDA-MB-468, and MDA-MB-231) of the tumor-suppressor protein p53 which was shown to enhance the activity of the mevalonate pathway when mutated in breast cancer^10,45,46^. Along those lines, MDA-MB-231 and MDA-MB-468 cells lack the ER, whereas MCF-7 and T47D cells are ER positive. These observations may point to a role of ER signaling in statin sensitivity and the mevalonate pathway feedback response upon HMGCR inhibition. However, the knockdown of the ER prior to statin treatment did not diminish HMGCR induction in MCF-7 cells (data not shown).

Recently, the expression of membrane E-cadherin was identified as a marker of statin-resistant tumor cells^25^. Future mechanistic studies may address the role of LDLR and SREBP-2 proteins as well as potential differences in external cholesterol dependency, cholesterol uptake potential, and post-translationally regulated HMGCR degradation in statin resistance of different human breast cancer cell types. It is also of great interest how long-term treatment with statins drives HMGCR activation as a specific mediator of statin resistance.

In conclusion, our results implicate that HMGCR expression is a key mediator of statin resistance in breast cancer cells that may result from an aberrant feedback loop within the mevalonate pathway. In addition, we demonstrated that statin-sensitive tumor cells can acquire statin-resistance following long-term exposure to statins. Targeting both the HMGCR by statins and its transcriptional regulation could be a useful tool to overcome statin resistance in tumor cells that warrant further investigation using respective in vivo models and primary tumor cells.

## Materials and methods

### Cancer cell lines and cell culture

The human breast cancer cell lines MDA-MB-231, MDA-MB-468, MCF-7, and T47D were obtained from ATCC (Manassas, VA, USA). MDA-MB-231, MCF-7, and T47D cells were cultured in DMEM/Ham´s F12 (Gibco Life Technologies, Darmstadt, Germany) and MDA-MB-468 cells in DMEM (Gibco Life Technologies, Darmstadt, Germany), supplemented with 10% fetal bovine serum (FBS; Biochrome, Berlin, Germany) and 1% penicillin/streptomycin (Gibco Life Technologies). Cells were grown in a humidified atmosphere of 95% air and 5% CO_2_. Short tandem repeat profiling of all used cell lines was performed in August 2017 at the DSMZ (German Collection of Microorganisms and Cell Cultures) to verify their genetic authenticity. MDA-MB-468 cells were newly acquired.

To establish a simvastatin-resistant MDA-MB-231 subclone, cells were persistently treated over a time period of 4–5 months with simvastatin starting with 2–5 µM and a stepwise increase in concentration up to 25 µM (Suppl. Fig. 1a). In the first weeks, treatment was performed as an “on/off” regimen, stopped when cells microscopically underwent apoptosis and restarted when remaining vital cells had recovered. Later, regimen was changed to a regular treatment of every 2–3 days and finally to a daily treatment with splitting of the cells on Fridays with no treatment over the weekend. Control cells were treated with respective DMSO concentrations using the same regimen. Experiments were started when the cells no longer showed any microscopic sign of apoptosis.

### Reagents and antibodies

The statins (mevalonate pathway inhibitors) used were simvastatin (SIM, Sigma-Aldrich, Hamburg, Germany), rosuvastatin calcium (ROSU, SelleckChem, Munich, Germany), and atorvastatin calcium salt trihydrate (ATO, Sigma-Aldrich, Hamburg, Germany). Stocks were prepared in DMSO. Primary antibodies for Western Blot analyses were: anti-Rap1A (sc-1482; recognizes the ungeranylated Rap1A^47^), anti-HMGCR (sc-271595) from Santa Cruz (Heidelberg, Germany), anti-HMGCR (ab214018) from Abcam (Cambridge, UK), anti-cleaved PARP (#9541) from Cell Signaling Technology, Inc. (Beverly, MA, USA), and anti-SREBP2 (#557037) from BD Biosciences (Heidelberg, Germany). For GAPDH, we used the anti-GAPDH (sc-25778) from Santa Cruz (Heidelberg, Germany) and anti-GAPDH (#5G4) from HyTest Ltd. (Turku, Finland). Secondary horseradish peroxidase-conjugated antibodies were anti-mouse IgG (HAF007), anti-rabbit IgG (HAF008), and anti-goat IgG (HAF109) from R&D Systems, Inc. (Minneapolis, MN, USA).

### RNA isolation, reverse transcription, and real-time PCR

The analysis of gene expression was performed as previously described^28,48^. Briefly, total RNA was isolated with the ReliaPrep^™^ RNA Tissue Miniprep System (Promega, Mannheim, Germany) according to the manufacturer´s instructions. Five-hundred nanogram RNA were reverse transcribed by using the M-MLV Reverse Transcriptase (Promega, Mannheim, Germany) and the complementary DNA (cDNA) was used for a SYBR green-based real-time PCR. A standard protocol was used for the analysis of gene expressions (Applied Biosystems, Carlsbad, CA, USA). The primer sequences (Sigma-Aldrich) for human genes were as follows: FDPS: CAGAATGAACGGAGACCAGA, GGGAGAAGTGCTGAACGAAA; *GAPDH* (glyceraldehyde 3-phosphate-dehydrogenase): AGCCACATCGCTCAGACAC, GCCCAATACGACCAAATCC; *HMGCR*: AGGAGGCATTTGACAGCACT, ACCTGGACTGGAAACGGATA; *LDLR*: GTGCTCCTCGTCTTCCTTTG, GTGGACCTCATCCTCTGTGG.

### Transfection with siRNAs

MCF-7 and T47D cells were grown until sub-confluence in 6-well or 12-well plates and transfected using DharmaFect and control siRNA (Silencer^®^ Select Negative Control #1 siRNA; Cat#4390843; Ambion, Life Technologies, Carlsbad, CA, USA) or gene-specific siRNA against HMGCR (#s141; Ambion, Life Technologies, Carlsbad, CA, USA). SiRNAs and DharmaFect were separately mixed with FBS-free Opti-MEM (Gibco Life Technologies, Darmstadt, Germany) and incubated for 5 min at room temperature (RT), pooled and incubated for 20 min at RT. After washing the cells with Hank's balanced salt solution, 850 µl Opti-MEM without penicillin/streptomycin were added. The transfection mixtures were added dropwise to each well (150 µl). Final concentration of the siRNAs was 100 nM. After 6 h, medium was changed to normal DMEM/F-12. Cells were directly treated or transferred into 96-well plates, allowed to adhere and then treated with different statins, respectively.

### Vitality, apoptosis, and staining assays of cancer cells

Cell viability was assessed using the CellTiterBlue^®^ assay (Promega, Mannheim, Germany). To measure apoptosis, a Cell Death ELISA (Roche) and a Caspase 3/7 Glo^®^ assay (Promega) were performed to detect DNA fragmentation and caspase activation within the cells. To visualize and quantify adherent cells, a crystal violet staining was performed: Cells were washed with phosphate buffered saline and fixed using 10% paraformaldehyde for 15 min at RT. Cells were washed with double-distilled water (ddH_2_0) and stained with crystal violet solution (0.02% in 2% ethanol) for 20 min at RT. Stained cells were intensively rinsed with ddH_2_0 and dried afterwards. The crystal violet dye was eluted with 10% sodium dodecyl sulfate (SDS) upon shaking. The absorbance was detected at 595 nm. Crystal violet and CellTiterBlue^®^ measurements were completed using the FluoStar Omega (BMG labtech, Jena, Germany).

### Immunoblotting

Western blot analyses were performed as described:^28^ briefly, total protein was isolated from treated cells by using a SDS-based lysis buffer (20 mM Tris/HCl pH 7.4; 1% SDS; protease inhibitor cocktail (Roche)). Twenty microgram of protein were loaded on a 10–12% SDS polyacrylamide gel electrophoresis. Separated proteins were blotted on 0.2 µm nitrocellulose membranes and blocked with 5% bovine serum albumin or 5% nonfat dry milk in Tris-buffered saline with 1% Tween-20 (TBS-T). Membranes were incubated with the primary antibodies in blocking buffer overnight at 4 °C, washed with TBS-T and incubated with the secondary antibody. Detection was performed using the luminescent ECL detection kit (Pierce, Thermo Fisher Scientific, Schwerte, Germany).

### Statistical analyses and software

Results are presented as means ± standard deviation. All experiments were repeated at least three times with independent biological replicates. For assessing cell vitality, apoptosis and gene expression, individual biological experiments were performed as duplicates, respectively. Outliers were determined via Grubb's test. Group analyzes were performed using one-way analysis of variance (ANOVA) by GraphPad Prism 6.07 (GraphPad, La Jolla, CA, USA). *p* Values < 0.05 were considered statistically significant. Final arrangement of the figures was performed using CorelDraw^®^ X6 version 16.0.0.707.

## Supplementary information

Supplementary Figure 1

Supplementary Figure 2

Supplementary Figure 3

Supplementary Figure 4

Supplementary Figure 5

Supplementary Figure 6

Supplementary Figure 7

supplemental figure legends
