# Supplementary figures and images for: Induction of 3-hydroxy-3-methylglutaryl-CoA reductase mediates statin resistance in breast cancer cells
Source: Cell Death Dis. 2019 Jan 28;10(2):91. doi: 10.1038/s41419-019-1322-x (PMC6349912; doi:10.1038/s41419-019-1322-x)

Suppl.Fig.1

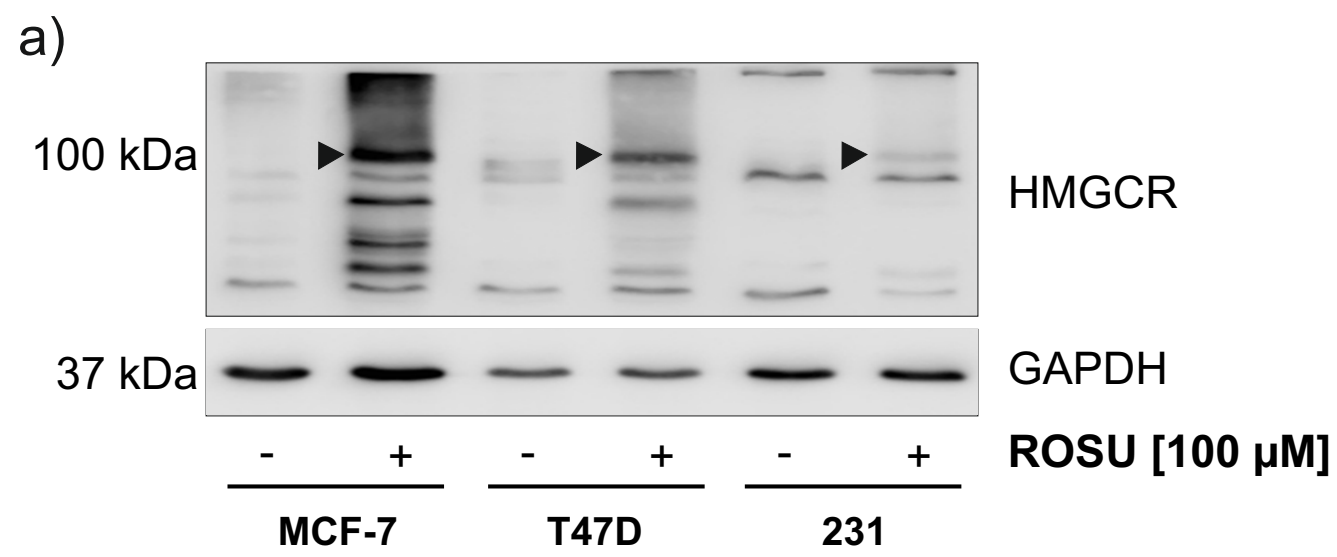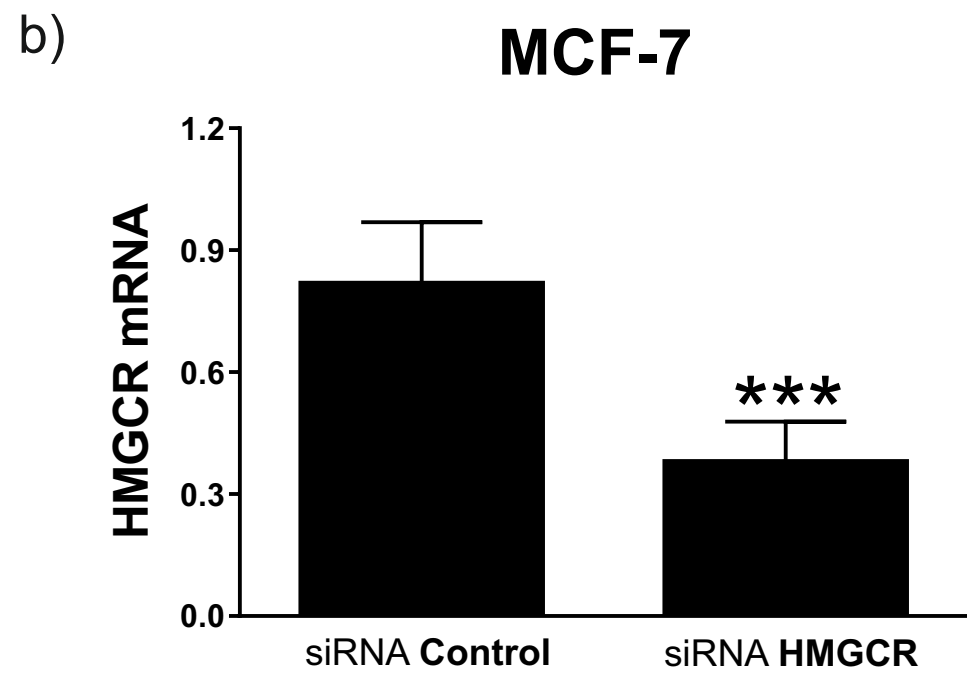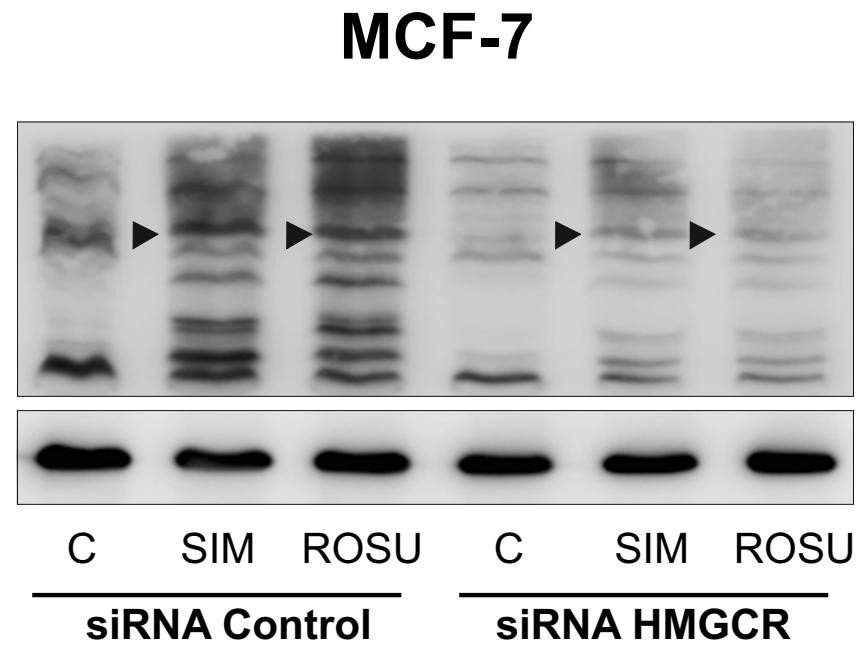

Supplement: Supplementary file 1 — Supplementary Figure 1 [file 41419_2019_1322_MOESM1_ESM.pdf]

# Suppl.Fig.2

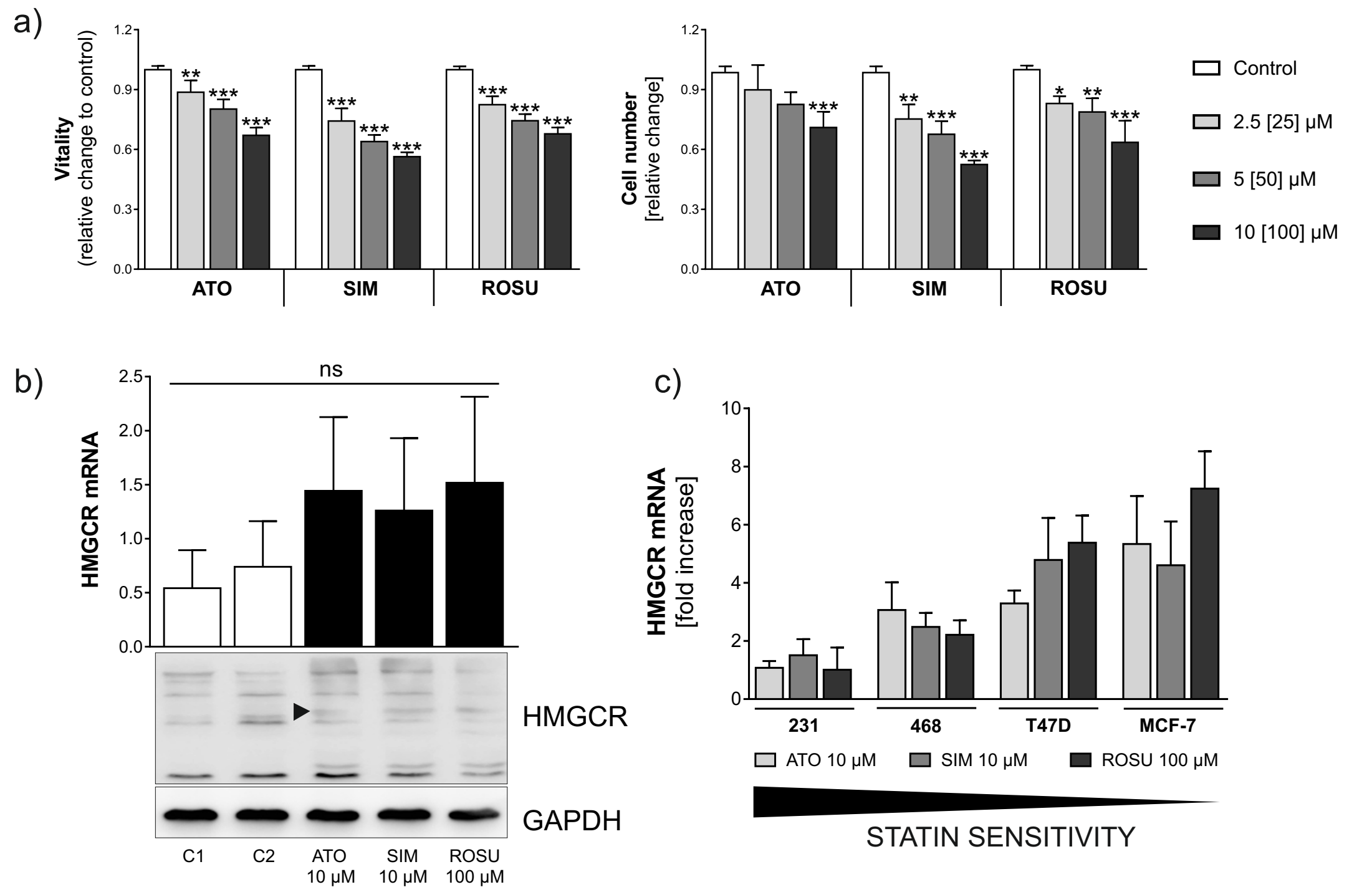

Supplement: Supplementary file 2 — Supplementary Figure 2 [file 41419_2019_1322_MOESM2_ESM.pdf]

Suppl.Fig.3

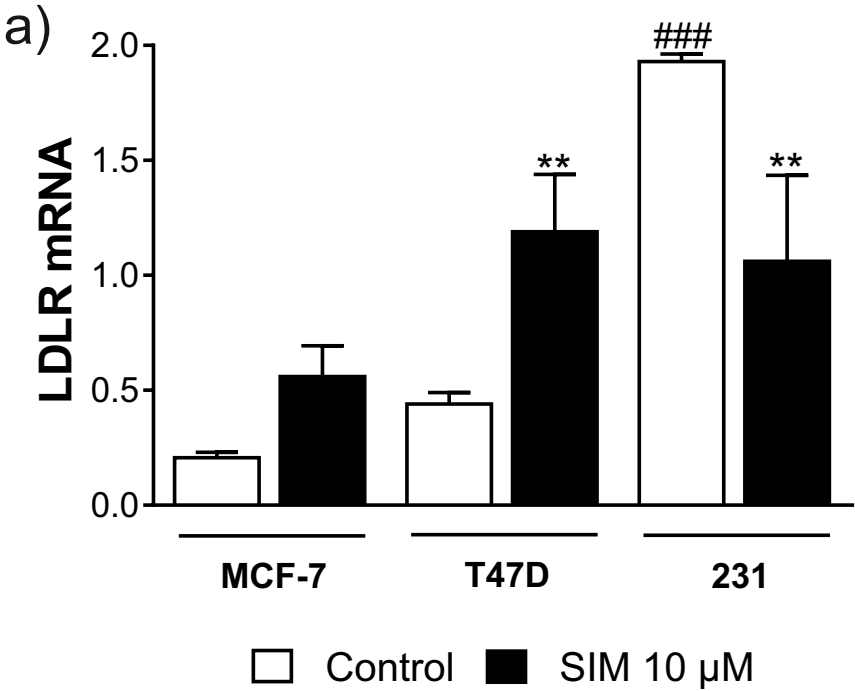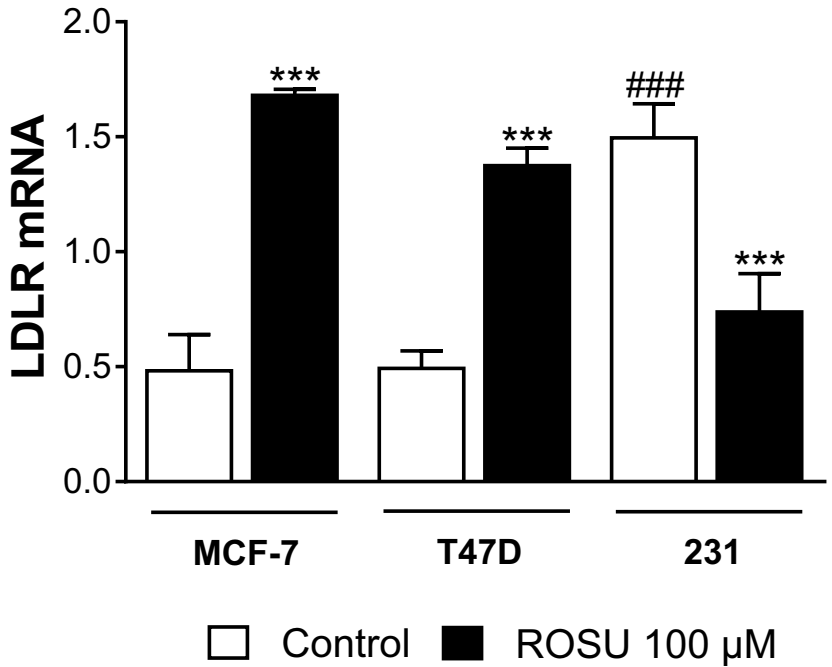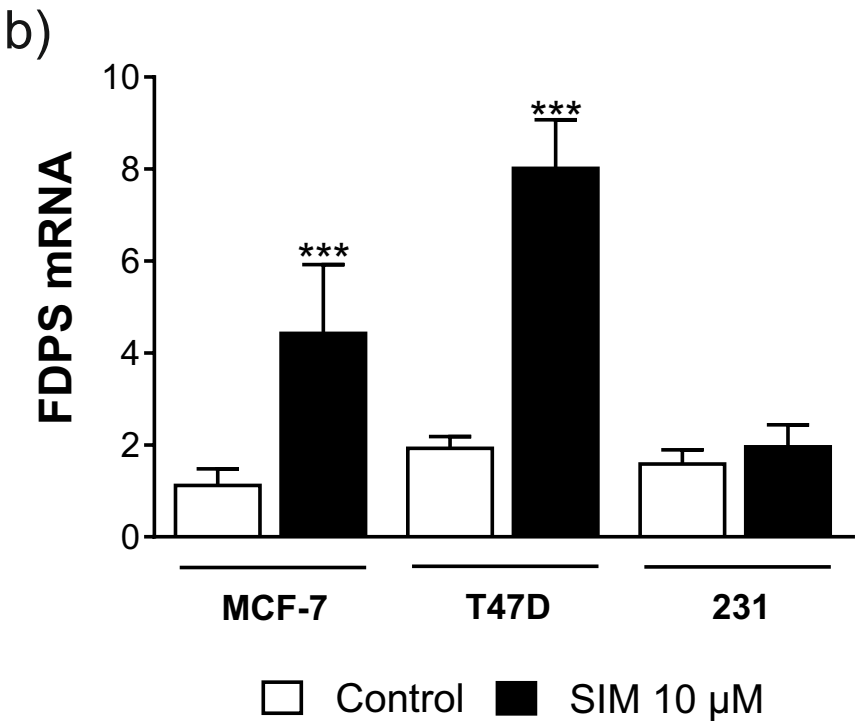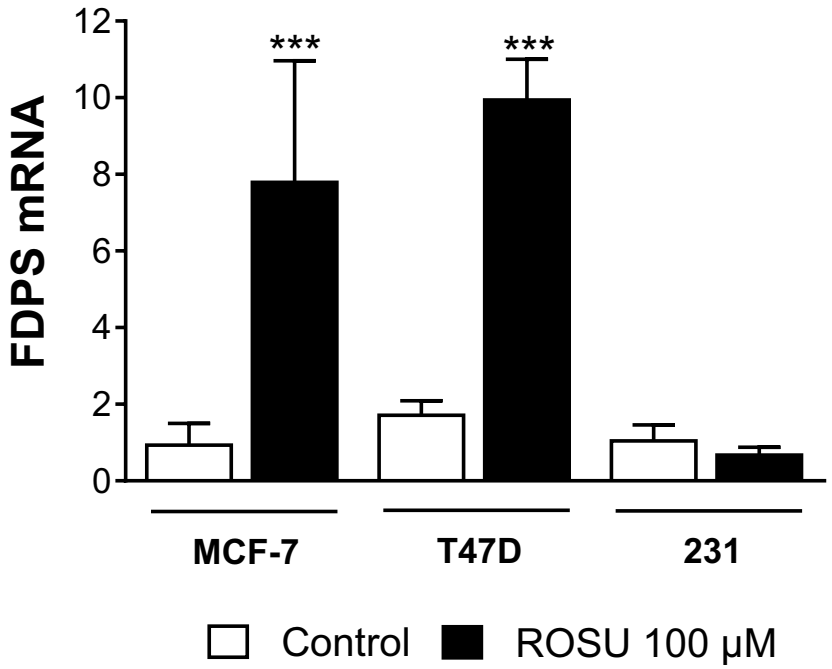

Supplement: Supplementary file 3 — Supplementary Figure 3 [file 41419_2019_1322_MOESM3_ESM.pdf]

# Suppl.Fig.4

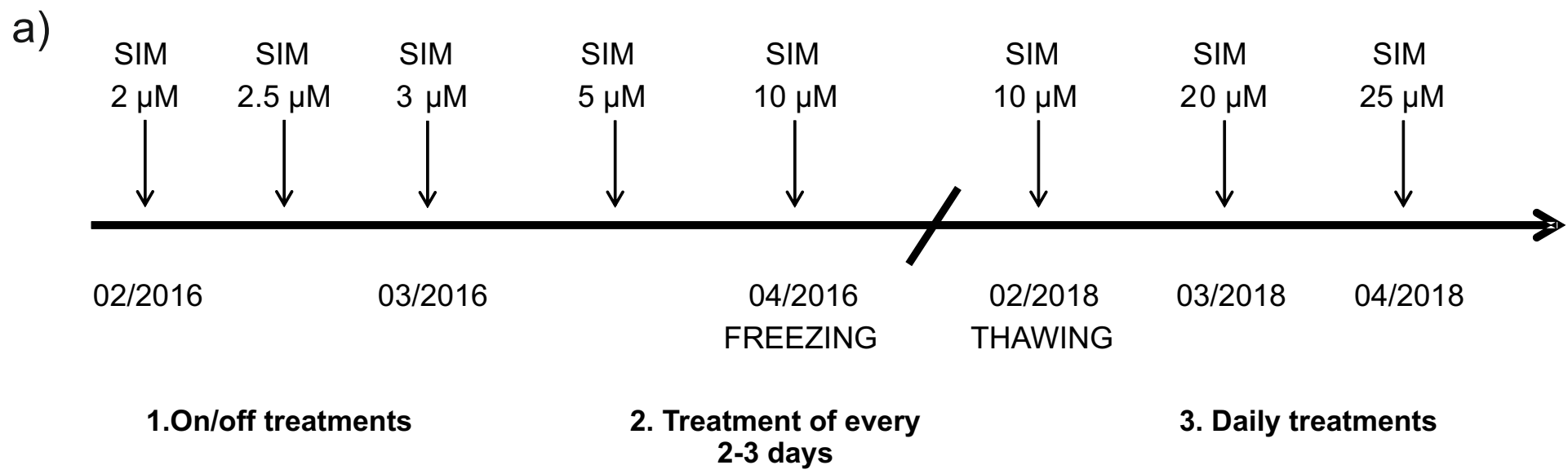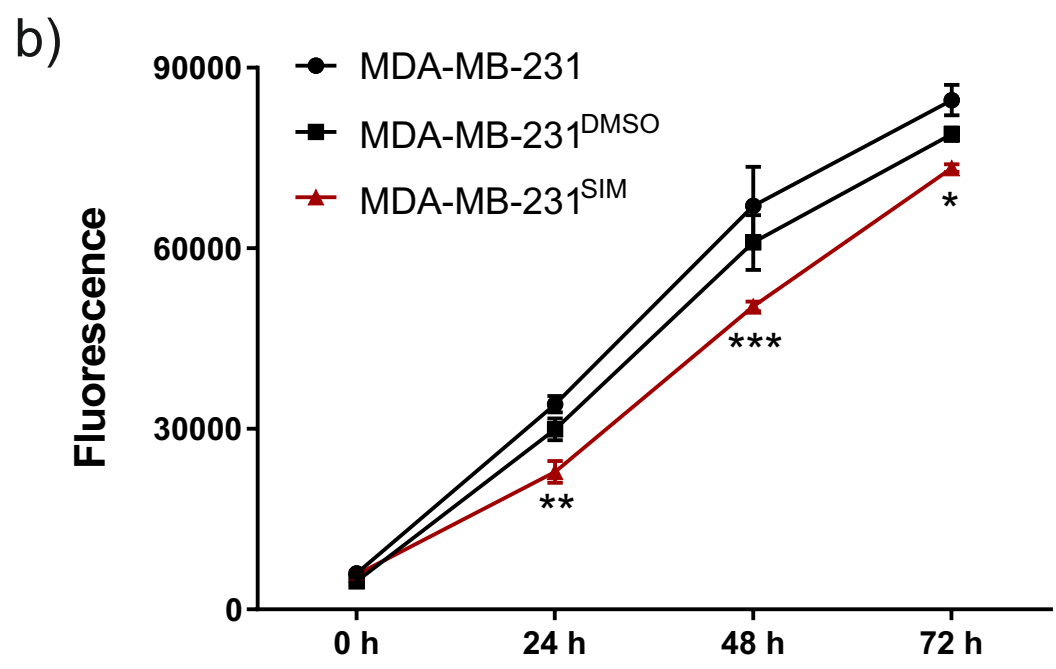

Supplement: Supplementary file 4 — Supplementary Figure 4 [file 41419_2019_1322_MOESM4_ESM.pdf]

Suppl.Fig.5

a)

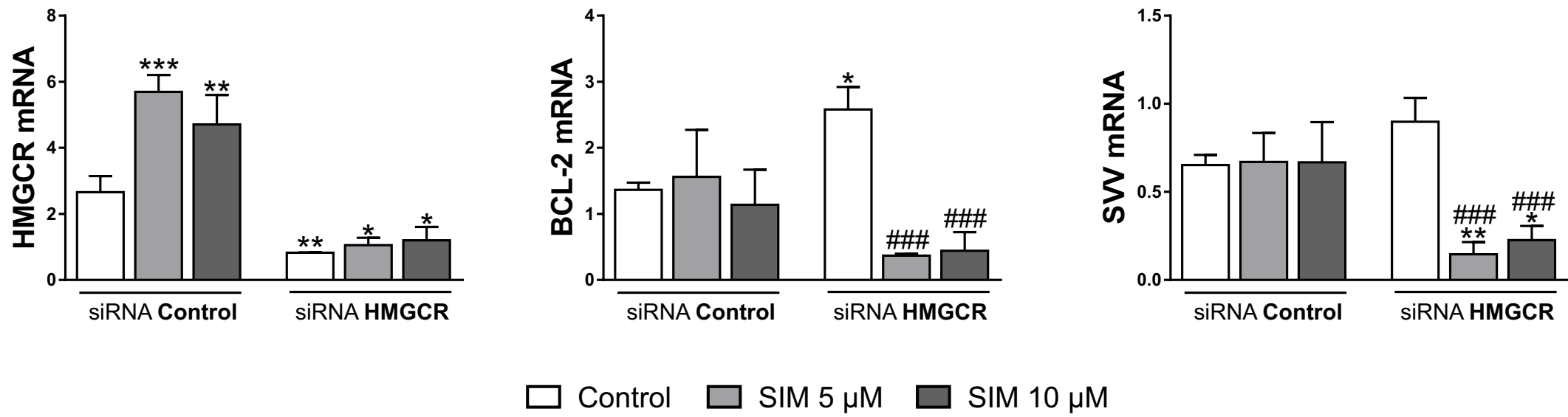

b)

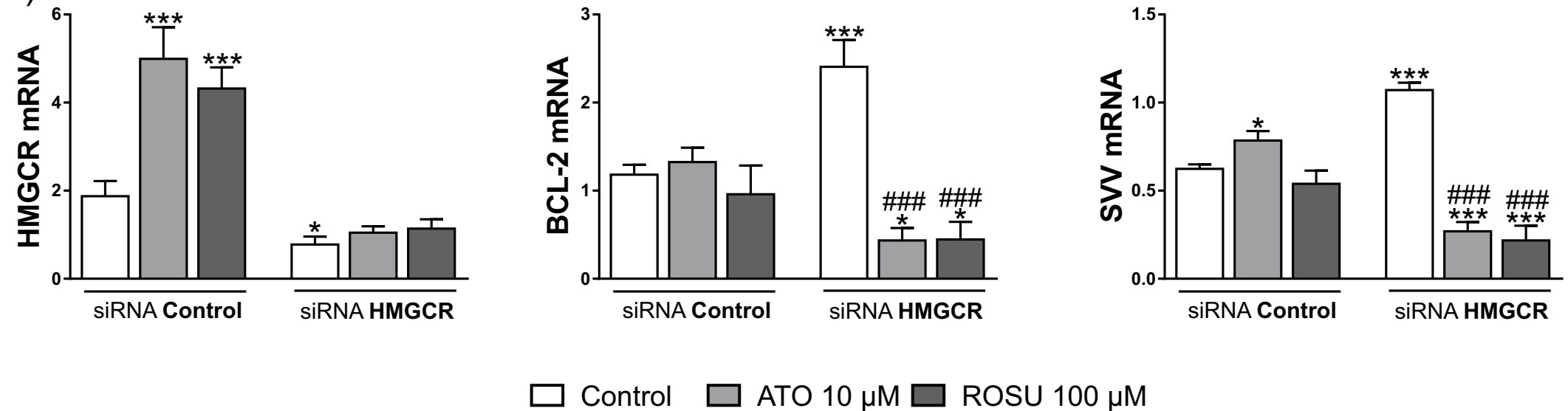

Supplement: Supplementary file 5 — Supplementary Figure 5 [file 41419_2019_1322_MOESM5_ESM.pdf]

Suppl.Fig.6

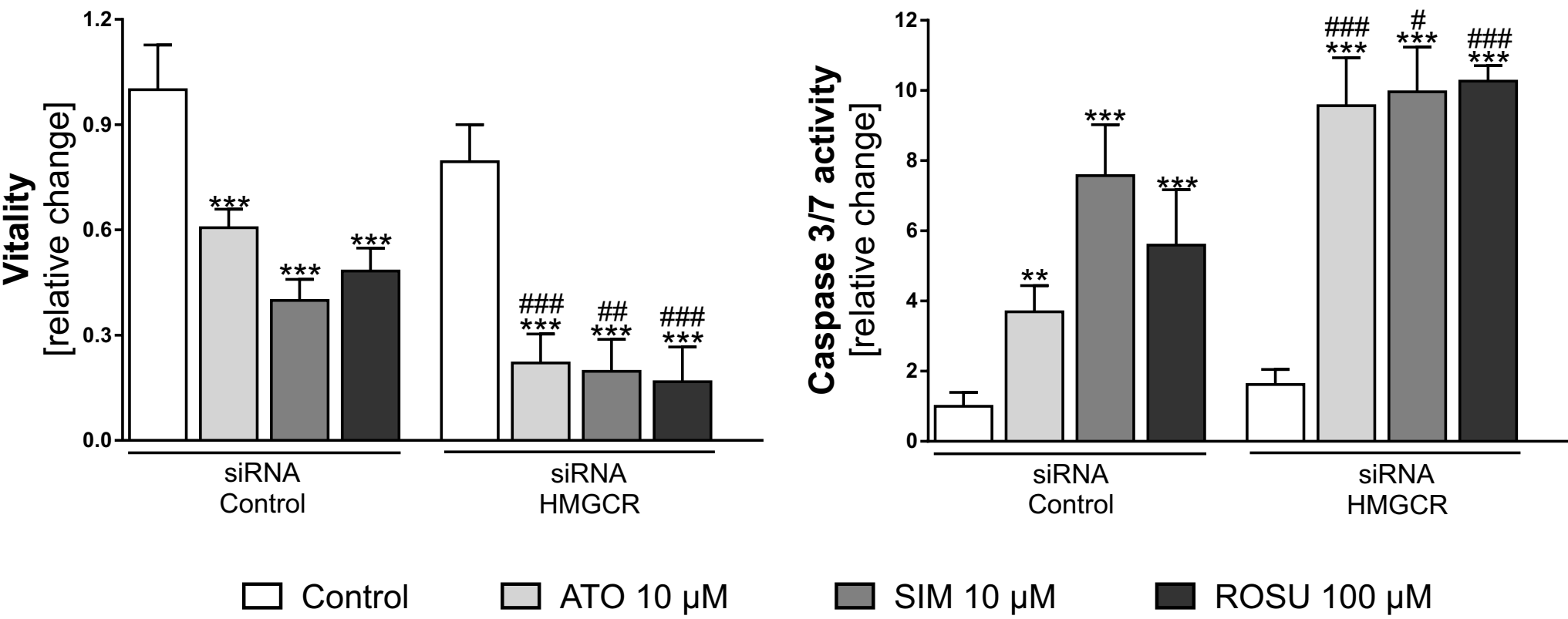

Supplement: Supplementary file 6 — Supplementary Figure 6 [file 41419_2019_1322_MOESM6_ESM.pdf]

Suppl.Fig.7

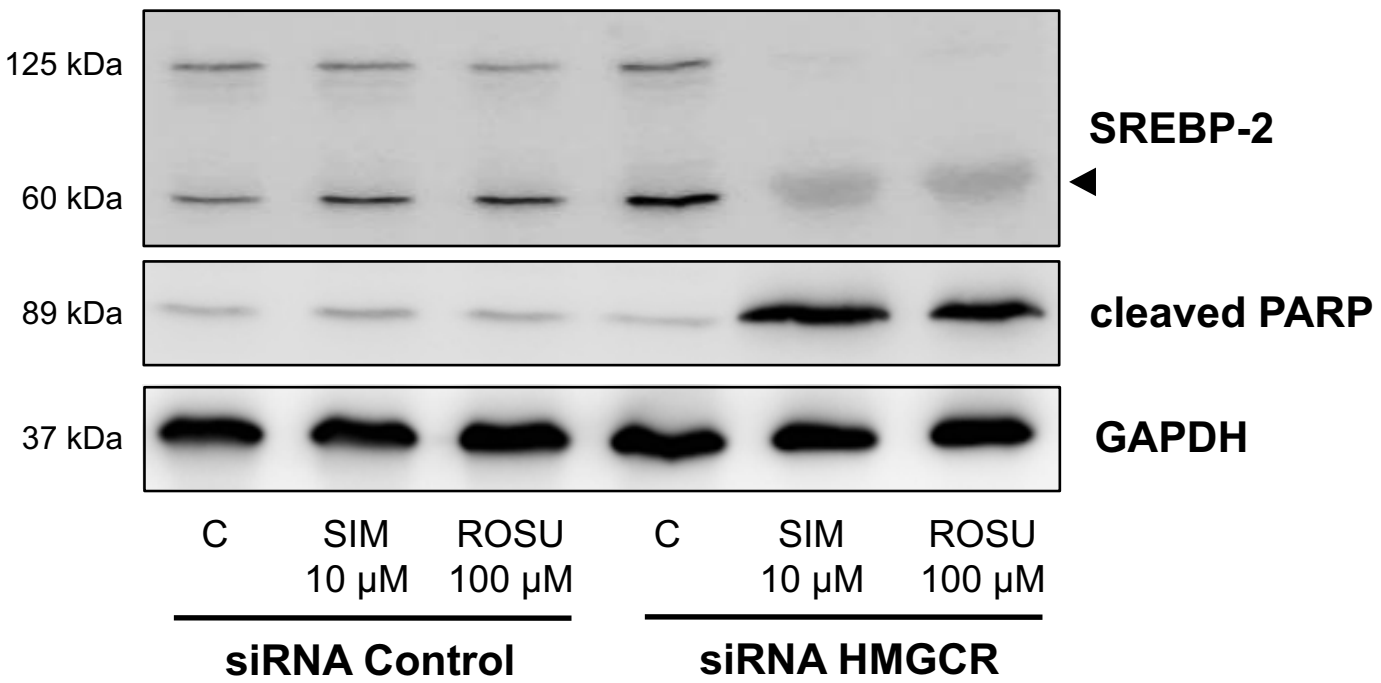

Supplement: Supplementary file 7 — Supplementary Figure 7 [file 41419_2019_1322_MOESM7_ESM.pdf]
