# Supplementary material for: Induction of 3-hydroxy-3-methylglutaryl-CoA reductase mediates statin resistance in breast cancer cells
Source: Cell Death Dis. 2019 Jan 28;10(2):91. doi: 10.1038/s41419-019-1322-x (PMC6349912; doi:10.1038/s41419-019-1322-x)
Supplement: Supplementary file 8 — supplemental figure legends [file 41419_2019_1322_MOESM8_ESM.doc]

**Supplementary figure legends**

**Suppl.Fig.1. Confirmation of 3-hydroxy-3-methylglutaryl-CoA (HMGCR) siRNA specificity and protein analyses**

Human MCF-7, T47D, and MDA-MB-231 breast cancer cells were treated with rosuvastatin (ROSU) for 48 h. Protein expression of HMGCR was investigated using western blot analysis (anti-HMGCR ab214018 from Abcam, **a**). Human MCF-7 cells were treated with HMGCR-specific and control siRNA. Knock-down was verified by real-time PCR (**b**). Data are shown as mean ± SD of at least three individual experiments. (***p ≤ 0.001) Cells were treated with statins 24 h after HMGCR knockdown for 48 h. Protein expression of HMGCR was investigated using Western blot analysis using ab214018 (**c**). Representative images are shown. The black arrowhead indicates the ~100 kDa size as the proposed protein size of the HMCGR. The additional bands point towards several isotypes and/or glycosylation status of the protein.

**Suppl.Fig.2. Human MDA-MB-468 are statin-sensitive and do not induce 3-hydroxy-3-methylglutaryl-CoA (HMGCR) gene expression**

MDA-MB-468 cells were treated with increasing concentrations of atorvastatin (ATO), simvastatin (SIM), and rosuvastatin (ROSU) for 48 h (concentrations for ROSU in brackets). The impact on cell vitality and cell number was measured using the CellTiterBlue® and the crystal violet assay (**a**). Gene and protein expression of the HMGCR were assessed using quantitative real-time PCR and Western blot analysis. Glyceraldehyde-3-phosphate dehydrogenase (GAPDH) was used a housekeeper control. Two different dimethyl sulfoxide controls were used: 1 : 1.000 (C1); 1 : 100 (C2) (**b**). All used breast cancer cell lines were arranged on the basis of their statin sensitivity according to the loss of vitality and post-statin HMGCR mRNA induction depicted (**c**). Data are shown as mean ± SD of at least three individual experiments. (*p ≤ 0.05; **p ≤ 0.01; ***p ≤ 0.001).

**Suppl.Fig. 3. Statin treatment increases low-density lipoprotein receptor (LDLR) and farnesyl diphosphate synthase (FDPS) gene expression in statin-resistant breast cancer cell lines**

Human MCF-7, T47D, and MDA-MB-231 breast cancer cells were treated with simvastatin (SIM), or rosuvastatin (ROSU) for 48 h. Gene expression of the LDLR (**a**) and the FDPS (**b**) was assessed using quantitative real-time PCR. Data are shown as mean ± SD of at least three individual experiments. *p ≤ 0.05; (**p ≤ 0.01; ***p ≤ 0.001).

**Suppl.Fig.4 Establishment of a simvastatin (SIM)-resistant MDA-MB-231 subclone**

MDA-MB-231 cells were persistently treated over a time period of four to five months with simvastatin starting with 2-5 µM and a stepwise increase in concentration up to 25 µM. In the first weeks, treatment was performed as an “on/off” regimen, stopped when cells microscopically underwent apoptosis and restarted when remaining vital cells had recovered. Later, regimen was changed to a regular treatment of every 2-3 days and finally to a daily treatment with splitting of the cells on Fridays with no treatment over the weekend. Control cells were treated with respective DMSO concentrations using the same regimen. Experiments were started when the cells no longer showed any microscopic sign of apoptosis (**a**). MDA-MB-231, 231^DMSO^, and 231^SIM-R^ cell vitality was measured after different time points using CellTiterBlue® assay (**b**). Data are shown as mean ± SD of at least three individual experiments. *p ≤ 0.05; (**p ≤ 0.01; ***p ≤ 0.001).

**Suppl.Fig. 5. Impact of a 3-hydroxy-3-methylglutaryl-CoA (HMGCR) knockdown on anti-apoptotic gene expression in statin-treated 231^SIM-R^ cells**

Human statin-resistant MDA-MB-231^SIM-R^ breast cancer cells were transfected with HMGCR-specific siRNA and treated with **a**) simvastatin (SIM) or **b**) atorvastatin (ATO) and rosuvastatin (ROSU) after 24 h for 48 h. Gene expression of the *HMGCR* and the anti-apoptotic genes B-cell lymphoma 2 (*BCL-2*) and surviving (*SVV*) was assessed using quantitative real-time PCR. Data are shown as mean ± SD of at least three individual experiments. Any treatments vs. untreated siRNA control: *p ≤ 0.05; **p ≤ 0.01; ***p ≤ 0.001; Treatments vs. untreated HMGCR siRNA: ### p ≤ 0.001

**Suppl.Fig. 6. Knockdown of 3-hydroxy-3-methylglutaryl-CoA (HMGCR) potentiates statin sensitivity of 231^DMSO^ cells**

Parental MDA-MB-231^DMSO^ breast cancer cells were transfected with HMGCR-specific siRNA and treated with atorvastatin (ATO), simvastatin (SIM) or rosuvastatin (ROSU) after 24 h for 48 h. Vitality and caspase 3/7 activation were measured using the CellTiterBlue® and Caspase 3/7 Glo® assay. Data are shown as mean ± SD of at least three individual experiments. Any treatment vs. untreated siRNA control: **p ≤ 0.01; ***p ≤ 0.001; treated HMGCR siRNA vs. untreated HMGCR siRNA: ^#^p ≤ 0.05; ^##^p ≤ 0.01; ^###^p ≤ 0.001 ).

**Suppl.Fig. 7. Knockdown of 3-hydroxy-3-methylglutaryl-CoA (HMGCR) sensitizes 231^SIM-R^ cells to simvastatin (SIM) and rosuvastatin (ROSU)**

Human statin-resistant MDA-MB-231^SIM-R^ breast cancer cells were transfected with HMGCR-specific siRNA and treated with SIM or ROSU after 24 h for 48 h. Protein expression of sterol regulatory element-binding protein (SREBP)-2 and cleaved poly ADP-ribose polymerase (PARP) was analyzed using Western blot. The arrowhead indicates the cleaved part of SREBP-2 protein. Representative images are shown.
